# Person-to-Person Transmission of Andes Virus

**DOI:** 10.3201/eid1112.050501

**Published:** 2005-12

**Authors:** Valeria P. Martinez, Carla Bellomo, Jorge San Juan, Diego Pinna, Raul Forlenza, Malco Elder, Paula J. Padula

**Affiliations:** * Instituto Nacional de Enfermedades Infecciosas ANLIS "Dr. C.G. Malbrán," Buenos Aires, Argentina; † Hospital de Infecciosas "F.J. Muñiz," Buenos Aires, Argentina; ‡ Hospital Privado de La Comunidad, Mar del Plata, Argentina; § Dirección de Epidemiología del Gobierno de la Ciudad de Buenos Aires, Buenos Aires, Argentina; ¶ Dirección de Epidemiología de la Provincia Neuquén, Neuquén, Argentina

**Keywords:** Andes virus, hantavirus, person to person transmission, Argentina, infectious source, virus spread, clusters, research

## Abstract

Despite the fact that rodents are considered to be the infectious source of hantavirus for humans, another route of transmission was demonstrated. Andes virus (ANDV) has been responsible for most of the cases recorded in Argentina. Person-to-person transmission of ANDV Sout lineage was described during an outbreak of hantavirus pulmonary syndrome in southwest Argentina. In this study, we analyzed 4 clusters that occurred in 2 disease-endemic areas for different ANDV lineages. We found new evidence of interhuman transmission for ANDV Sout lineage and described the first event in which another lineage, ANDV Cent BsAs, was implicated in this mechanism of transmission. On the basis of epidemiologic and genetic data, we concluded that person-to-person spread of the virus likely took place during the prodromal phase or shortly after it ended, since close and prolonged contact occurred in the events analyzed here, and the incubation period was 15–24 days.

## Abstract 2

Epidemiologic and genetic data show that person-to-person spread likely took place during the prodromal phase or shortly after it ended.

The genus *Hantavirus* is a growing group of rodentborne viruses of worldwide distribution that cause human diseases. *Hantavirus* is the only genus of the family *Bunyaviridae*, which comprises rodentborne viruses. Specific species of rodents are natural reservoirs for different hantavirus types. In America, hantaviruses are mainly carried by sigmodontine rodents. Hantavirus pulmonary syndrome (HPS) was first described in North America in 1993 (*1**,**2*), and then reported in several other countries of North, Central, and South America (*3**–**9*). Andes virus (ANDV) was characterized in Argentina in 1995 on the basis of specimens from a patient who died of HPS (*3**,**10*), and Andes virus has been responsible for most HPS cases recorded in Argentina, Chile, and Uruguay (*7*).

Six different ANDV lineages have been reported to cause HPS in Argentina: ANDV Sout in the southwest region; ANDV Cent BsAs, ANDV Cent Lec, and ANDV Cent Plata in the central region; and ANDV Nort Orán and ANDV Nort Bermejo in the northwest region. The definition of these 6 lineages was previously established on the basis of nucleotide and amino acid differences (*7**,**9*). Although rodents are considered to be the infectious source for humans, another route of infection was demonstrated. Viral person-to-person transmission of ANDV Sout lineage was described for the first time during an HPS outbreak in southwest Argentina in 1996, in which 16 persons were involved (*11**–**13*), but in general, clusters of HPS cases are mainly attributed to a common source of rodent exposure. This mechanism of interhuman virus spread, which makes ANDV unique among the hantaviruses, is not the only exclusive feature of this virus. ANDV was the only American hantavirus isolated from human serum (*14*). Most importantly, ANDV was shown to be highly lethal in Syrian hamsters, and the characteristics of the disease closely resembled HPS in humans (*15*). Similar results have been recently obtained with Maporal virus (*16*).

We analyzed 4 case clusters of hantavirus infection and present new evidence for interhuman transmission of ANDV Sout lineage. We also describe the first event in which another lineage, ANDV Cent BsAs, has been implicated in this rare but already proven route of transmission.

## Materials and Methods

### Study Population

Thirteen HPS cases that occurred during the second half of 2002 in Argentina were analyzed in this study (Table 1). Cases were grouped in 4 clusters (C1–C4) according to their known epidemiologic relationships. Samples from 12 of the 13 case-patients were available: we collected serum and clot samples from almost all patients; from patient C4-a, clot samples could not be obtained; from patient C2-d1, a hemoculture was available after death. A sample from the son in cluster 2 (C2-s) was not available. Serologic confirmation was performed on 11 serum specimens and on the hemoculture samples as previously described (*17*).

**Table 1:** Hantavirus pulmonary syndrome patients and their epidemiologic relationships, Argentina, 2002*

Cluster/patient	Age, y	Date of onset	Date of death	Residence	Probable place of infection or risk activity
C1-f	41	7/10/02	7/16/02	BA City	Farm in LP (BA)
C1-s	14	8/8/02		BA City	Spent weekend at his father's house (Jul 13–14/02)
C2-d1	12	7/24/02	7/29/02	LP, rural area, (BA). El Peligro neighborhood.	Home
C2-d2	11	7/28/02		LP, rural area, (BA). El Peligro neighborhood.	Home
C2-s	NA			LP, rural area, (BA). El Peligro neighborhood.	Home
C2-m	40	8/04/02	8/15/02	LP, rural area, (BA). El Peligro neighborhood.	Home
C3-1	28	8/26/02		LP, rural area, (BA). Abasto neighborhood.	Home
C3-2	27	8/29/02		LP, rural area, (BA). Abasto neighborhood.	Home
C3-3	21	9/10/02		LP, rural area, (BA). Abasto neighborhood.	Home
C3-4	30	9/14/02		LP, rural area, (BA). Abasto neighborhood.	Home
C4-a	39	11/21/02		CI (RN)	Visited VA (NQ), Oct 20–30/02
C4-b	58	12/08/02		MP (BA)	Traveled by bus with C4-a from MP to NQ (11/23/02)
C4-c	38	12/31/02	1/02/03	MP (BA)	Several contacts with C4-b
NL-1	17	10/27/98		LP (BA)	Rural worker
NL-2	42	11/7/02	11/14/02	VA (NQ)	Rural worker. bitten by a rodent 9 days before the onset of symptoms
NL-3	22	12/8/02		VA (NQ)	Rural worker
NL-4	31	12/19/97		LO (NQ)	Housekeeper
NL-5	56	12/02/99	12/08/99	SM (NQ)	Rural worker

*BA, Buenos Aires; NL, nonlinked cases; LP, La Plata City; NA, not available; CI, Cipolletti City; RN, Rio Negro Province; NQ, Neuquén; MP, Mar del Plata City; VA, Villa La Angostura City; LO, Loncopué City; SM, San Martín de los Andes City; NA, not available.

### Genetic Characterization

Ten clot samples and 1 serum sample were subjected to viral detection methods as described (*7*). For viral genetic characterization, a partial fragment of G2-encoding region from the M segment was analyzed: genomic positions 2717–2943 (G2). For cases sharing 100% nucleotide identity in G2 fragment, 1 or 2 additional regions were analyzed: positions 66–434 from G1-encoding region (G1); positions 1384–1795 for C4 and 1395–1809 for C1 from the S-noncoding region (S-NCR). All fragments were numbered in the antigenome-sense sequence relative to ANDV. The new sequences used in this manuscript have been submitted to GenBank (accession nos. DQ189092–DQ189095).

## Results

### Epidemiologic Findings

**Figure 1:**
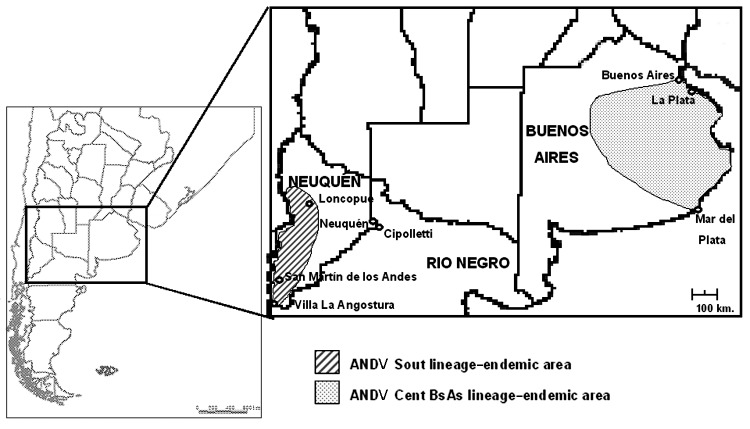
Location of residences and possible sites of exposure of hantavirus pulmonary syndrome patients in the provinces of Buenos Aires and Neuquén and Andes virus (ANDV)–endemic regions, 2002.

**Figure 2:**
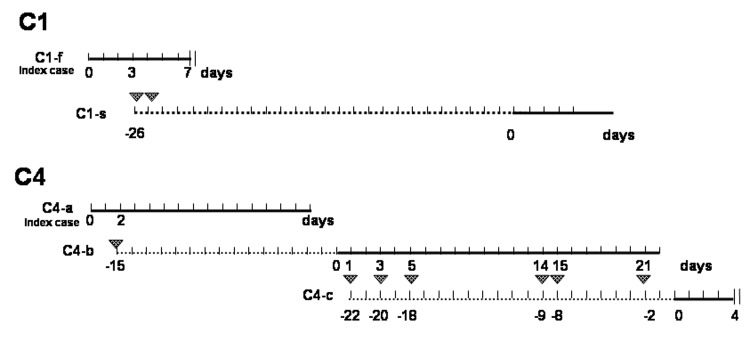
Contact events and incubation period of hantavirus pulmonary syndrome patients, Argentina, 2002. One line was drawn per case for C1 and C4; dotted lines represent the incubation period since the established moment of contact between contiguous case-patients. The onset of illness for each patient is indicated by day 0. Triangles indicate contacts between patients. | | indicates day of death.

The cases were grouped into 4 clusters (Table 1), and the approximate geographic locations of the exposure sites are shown in Figure 1. Cluster 1 (C1) was a father-son pair (C1-f and C1-s). The first patient, C1-f, was a previously healthy veterinarian. He worked on a farm >65 km away from his home. He began working there 40 days before the onset of symptoms. He slept at the farm during the week and returned to his home on weekends. The first manifestation of his illness, abdominal pain and vomiting, began on a Wednesday. On the Friday of that week, he exhibited typical indistinguishable features of the prodromal phase. The following weekend, he remained in his house with his 2 sons. Although he was very ill, his younger son slept with him in the same bed; at that time, the prodromal phase was ending, since on the next Monday he began to experience progressive dyspnea. He sought medical attention but proceeded rapidly to pulmonary edema and shock. He died on Wednesday. His 2 sons had never visited his place of work, and they lived with their mother in another house in the city of Buenos Aires. Epidemiologist from Buenos Aires City reported that both houses were in urban areas, were in very good condition, and showed no evidence of rodents. Twenty-four days after the last contact with his sick father, fever developed in the younger son (C1-s) (Figure 2). Once immunoglobulin M antibodies to hantavirus was confirmed, he was hospitalized.

Cluster 2 (C2) was a rural cluster within a family of 5 that lived in a cabin. Rodent infestation of the house and surrounding areas was evident. The onset of symptoms of 3 of the 4 case-patients occurred during a 12-day period (Table 1). The mother in C2 (C2-m) began having symptoms when HPS was already confirmed in her 2 daughters (C2-d1 and C2-d2), but she did not seek medical consultation until 5 days later, when she was hospitalized and died.

Cluster 3 (C3), a rural cluster composed of 4 friends (cases C3-1 to C3-4), was similar to the previous cluster since all became ill during a period of 20 days (Table 1). They lived together in a cabin within the farm where they worked. Rodent infestation of the house and surrounding areas was also evident.

Cluster 4 (C4) involved a case-patient (C4-a) and a person he came into contact with (C4-b), and a third person who came into contact with C4-b. The index patient lived and worked in Neuquén City, where neither HPS cases nor rodent infestation had been registered. He traveled to Mar del Plata in the province of Buenos Aires, 1,005 km northeast of Neuquén City, where he remained for a few days while attending a meeting. His first symptoms (fever and myalgia) began before his return to Neuquén from Mar del Plata, 23 days after he returned from a vacation in Villa La Angostura, a small town surrounded by a wilderness area, 447 km southwest of Neuquén City (Figure 1). Two HPS cases were reported in 2002, and several infected rodents were captured previously. During the case-patient's 14-hour bus trip from Mar del Plata to Neuquén, he sat next a man he did not know (C4-b). During the trip, a nonproductive cough, dyspnea, tachypnea, and headache developed, and myalgia worsened. After their arrival in Neuquén, patient C4-b helped patient C4-a with his luggage and shared a taxicab with him. Patient C4-a was hospitalized 24 hours later. Patient C4-b spent 2 days in Neuquén (he never left the city). Fifteen days after the 14-hour bus trip to Neuquén City, patient C4-b became ill. He first experienced weakness and myalgia; vomiting and diarrhea developed 2 days later, and fever developed 6 days after the onset of the first symptoms. C4-b met a friend and co-worker (C4-c) 3 times during his prodromal phase; C4-c visited C4-b while he was hospitalized (Figure 2). Although C4-b did not have respiratory clinical symptoms, the chest radiograph showed interstitial infiltrates and Kurley B lines, and he had severe hypoxemia. The major clinical manifestations were weakness, gastrointestinal illness, and myalgia. An epidemiologic investigation led clinicians to suspect HPS. C4-c began to exhibit the first HPS symptoms 22 days after C4-b. C4-c had never left the province of Buenos Aires.

### Viral Characterization

Nucleotide sequence comparisons of G2 fragment between sequences from the 11 cases, 5 nonlinked (NL) cases, and 5 previously published ANDV lineages are shown in Table 2. Comparisons in each of the 4 clusters showed 100% identity between cases in the same cluster. ANDV Cent BsAs lineage was characterized from C1, C2 (2/4 cases), and C3, while ANDV Sout was characterized from the 3 C4 cases, although C4-3 had never been to the southwestern part of the country. The 3 clusters from La Plata and NL-1 showed the highest similarity between them and showed significant differences with HPS cases from other places in Buenos Aires (Table 1).

**Table 2:** Andes virus (ANDV) lineage identification by comparison of nucleotide sequence identity percentages between hantavirus pulmonary syndrome clustered cases and previously reported ANDV lineages*

	ANDV Cent Plata	ANDV Cent Lech	ANDV Cent BsAs	ANDV Sout	ANDV Nort Orán	ANDV Nort Berm	C1-f/s	C2-d1/m	C3-1/2/3	C4-a/b /c	NL-1	NL-2	NL-3	NL-4	NL-5
ANDV Cent Plata		87.61	82.3	84.96	85.4	85.84	80.53	80.97	80.97	83.19	81.42	83.19	85.4	82.74	82.74
ANDV Cent Lech			82.74	82.3	84.51	88.05	83.19	82.74	82.74	80.53	84.07	80.53	82.74	80.9	81.42
ANDV Cent BsAs				80.97	84.07	81.86	94.69	95.58	95.58	83.3	95.13	83.3	81.42	81.42	81.86
ANDV Sout					84.86	80.97	81.42	80.97	80.97	96.02	81.86	96.02	98.67	94.69	95.58
ANDV Nort						85.84	84.07	82.74	82.74	82.3	84.07	82.3	85.4	85.4	81.86
ANDV Berm							82.3	81.42	81.42	80.09	82.74	80.09	82.3	82.3	79.65
C1-f/s								97.35	97.35	82.74	97.79	82.74	82.3	81.86	82.3
C2-d1/m									100	82.3	98.67	82.3	81.86	81.42	81.86
C3-1/2/3										82.3	98.67	82.3	81.86	81.42	81.86
C4-a/b/c											83.19	100	95.58	93.36	99.56
NL-1												83.19	82.74	82.3	82.74
NL-2													95.58	93.36	99.56
NL-3														95.13	95.13
NL-4															92.92
NL-5															

*Comparisons were made on 226 nucleotides from the G2-coding region.

### Assessment of the Route of Transmission

Rodent transmission is the most common route of hantavirus infection. However, when a new HPS case is suspected, if rodent exposure was not evident, for interhuman transmission to be suspected, one must 1) find a epidemiologic link with a previous case-patient (index case-patient); 2) confirm 100% viral nucleotide identity with the index patient in the fragments analyzed; and 3) assuming that the secondary patient could have been exposed to infectious rodents in the same or a different place than the index patient, determine the probability that both viral strains have 100% nucleotide identity in the fragments analyzed.

In C1, only C1-f had an evident rodent exposure. The only risk for C1-s was the close contact with C1-f during his prodromal phase. In C4, only C4-a had an evident risk of rodent transmission because he had visited a disease-endemic area. The only risky activity of C4-b was close contact with C4-a during the 14-hour bus trip. C4-c was in contact with C4-b at several times after the latter returned from Neuquén. All nucleotide fragment comparisons showed 100% identity for G1, G2, and S-NCR for C1 and C4. We analyzed viral variability in the areas of circulation of AND Cent Bs As and ANDV Sout lineages by comparing viral nucleotide sequences from previous HPS cases and found a positive correlation between geographic distance and genetic distance for each ANDV lineage (data not shown). Each ANDV lineage showed a significant degree of variability between the supposed sites of exposure in C1 for C1-f and C1-s and in C4 for C4-a and C4-b. In summary, C1-s, C4-b, and C4-c were infected by interhuman transmission.

## Discussion

From July to December 2002, 31 HPS cases were reported in Argentina, 13 of which were included in this study because they occurred as linked cases grouped in 4 clusters. Three of these clusters occurred in the province of Buenos Aires, where previous cases were isolated and sporadic (*18*). This is the first report of grouped HPS cases in this province. In contrast, such an occurrence is not rare in southern Argentina, where several clusters have been detected since the first HPS case was described in 1995. Three of these previous clusters occurred in the province of Neuquén. In the present study, we described 3 events of interhuman transmission: C1-f to C1-s, C4-a to C4-b, and C4-b to C4-c. Besides the complete identity of the fragments analyzed in each cluster, we conclude this mechanism took place based on the following facts: for C1, only C1-f was exposed to rodent infection in his work place (La Plata), and C1-s did not visit C1-f's work place. Similarly, in C4, C4-a was the only one of the 3 case-patients with epidemiologic risk of infection by rodent exposure during his vacation in Villa La Angostura. C4-b spent 2 days in Neuquén City, where HPS cases have never been reported. C4-c, who never left the province of Buenos Aires, could not have been infected by an endemic lineage from a place 1,300 km distant. Furthermore, 100% nucleotide identity was found between the C4 strain and NL-2, which confirmed the hypothesis that C4-a was exposed to ANDV in Villa La Angostura. In conclusion, C4 showed 2 links of the same chain of transmission. Notably, short or long periods between patients' onset of illness probably correspond to clusters with a common source of infection (C2) or to the occurrence of human transmission (C1 and C4) (Figure 2). The identical viral sequence in different HPS cases might be explained by exposure to the same viral variant within the local rodent populations, or in special situations, by virus spread from person to person. Genetically differentiating between these 2 mechanisms of transmission is not possible in a small area, as in C2 and C3. However, geographically distant patients rarely share identical viral sequences; in these situations, if epidemiologic relationships between patients are shown, suspecting interhuman transmission is reasonable, as in C1 and C4.

This is the first report in which a lineage other than ANDV Sout, that is, ANDV Cent BsAs, was implicated in person-to-person transmission. This finding is relevant because ANDV Cent BsAs was responsible for most HPS cases in the province of Buenos Aires (*18*). Furthermore, the possibility that the other lineages can be spread by this mechanism cannot be discarded, and such an event could be expected in any of the 3 affected regions. A well-done epidemiologic investigation around each case is essential to accurately establish the mechanism of infection. The incubation period was 15 days for C4-b, and ranged from 24 to 26 days for C1-s and 18 to 22 days for C4-c. However, the oldest son of C1-f did not become infected, and taking into account that C1-s rested with C1-f in the same bed during the end of the prodromal phase, we speculate that close and prolonged contact is necessary to produce the infection. In C4, a unique close contact was established between 2 previously unknown persons, C4-a and C4-b, while C4-a exhibited early clinical manifestations of respiratory disease. In this event, the unique route of transmission was by means of small-particle infectious saliva or respiratory aerosols during the close contact between both persons, since C4-b was the only passenger of the bus in whom the illness developed. Recent experimental studies with sigmodontine rodents hosting ANDV investigated the hypothesis that saliva was one of the sources of infection within reservoir populations (*19*).

In summary, virus transmission from one person to another likely occurs during the prodromal phase or shortly after it ends. Previously published reports of person-to-person transmission did not provide details of clinical manifestation at the moment of contact, but several infections occurred while some HPS patients were initially hospitalized; other patients contracted the infection after being in contact with a recently symptomatic case-patient (*12*). Current evidence indicates that casual contacts with a person in early stages of HPS are not risky, but special consideration should be given to contacts with case-patients that occurred in confined places such as within vehicles or some work environments.

Since interhuman transmission of ANDV occurred in Argentina in 1996, the major question that clinicians have been facing is what to do about personal protection and patient isolation. The answer to physicians likely to encounter HPS patients is to follow the recommended universal precautions. However, the findings presented here suggest that the most probable period of virus spread would be during the days before medical attention is sought. For this reason, the family or those who had close contact with an HPS patient during the prodromal phase seem to have more risk of interhuman infection than do clinicians. In the management of contacts of HPS cases caused by ANDV, epidemiologists usually give more relevance to serologic tests than to clinical surveillance. Whether serologic tests on specimens from asymptomatic contacts are worthwhile should be determined. Such results are usually misinterpreted since a negative serologic test result does not mean that the contact could not be incubating the infection, and a few days later, HPS symptoms could develop. This negative serologic result usually leads to delay in medical consultation. In our opinion, a strict clinical surveillance would be more valuable: contacts of HPS patients need to be monitored and advised to immediately look for medical evaluation as soon as fever or any other prodromal symptom develops. In this circumstance, a fast laboratory diagnosis is essential so virus spread can be avoided and early intensive care and treatment initiated. Patients with a confirmed diagnosis should be transferred to a unit skilled in intensive cardiopulmonary care. A previous study suggests that the earlier a patient is hospitalized, the higher the probability of survival (*20*). Unfortunately, effective vaccines, immunotherapeutic agents, and antiviral drugs for the prophylaxis or treatment of hantaviral infections are not available (*21*); thus far, results have been inconclusive regarding the usefulness of intravenous ribavirin in treating HPS (*22*).

Further studies will be necessary to understand more about this rare mechanism of virus spread. More information of virus variability of all the ANDV lineages will also help differentiate an instance of common rodent exposure from a new event of person-to-person transmission, especially in patients with a travel history.
